# Intravesical Protrusion of a Pedunculated Colonic Polyp through a Sigmoido-Vesical Fistula Caused by Diverticulitis

**DOI:** 10.5334/jbsr.1874

**Published:** 2020-01-28

**Authors:** Olivia del Marmol, Bruno Coulier, Frédéric Pierard

**Affiliations:** 1 Clinique Saint-Luc, Bouge, BE

**Keywords:** Colo-vesical fistula, sigmoid, diverticular disease, colonic polyp

## Abstract

**Teaching Point:** Sigmoid diverticulitis may cause colo-vesical fistula from which intravesical protrusion of a pedunculated colonic polyp is an exceptional event that should not be regarded as a bladder cancer.

## Case Report

**Figure 1:**
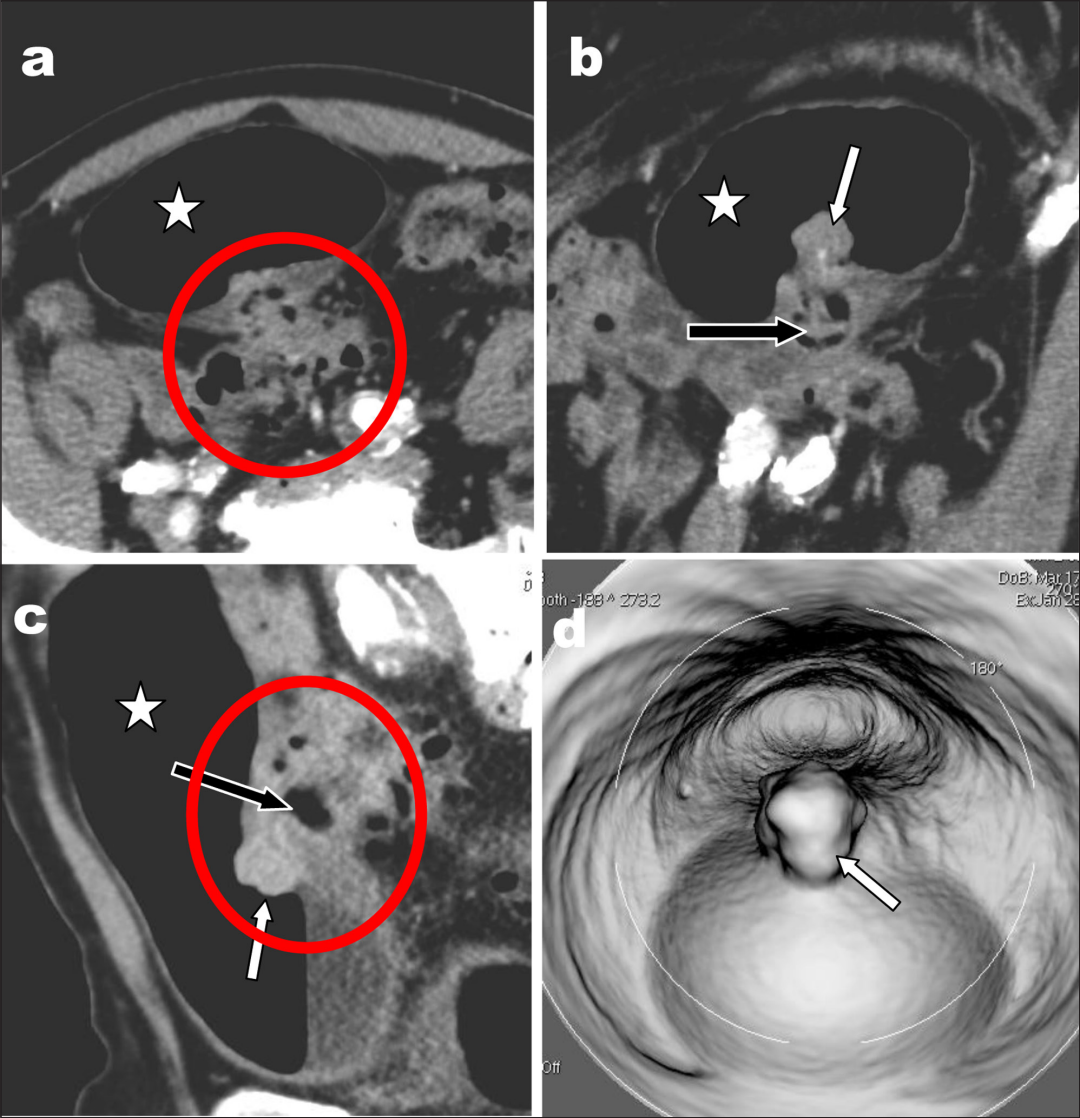


A 72-year-old male, recently treated for recurrent lower urinary infection, was admitted with fecaluria and pneumaturia. Contrast-enhanced abdominal computed tomography (CT) (Figure 1a–d) found a massive amount of gas within the bladder (white star) and loss of anatomic demarcation between the sigmoid colon and the posterior bladder wall, which respectively displayed diverticula and thickening. A large colo-vesical complex (within red circle) likely complicating sigmoid diverticular disease (black arrow) was protruding through the bladder wall. Additionally, a 1.5 cm unusual contrast enhancing mass (white arrow) was protruding within the bladder at the tip of the colo-vesical fistulous complex on axial (b) coronal (c) and virtual cystoscopy (d).

**Figure 2:**
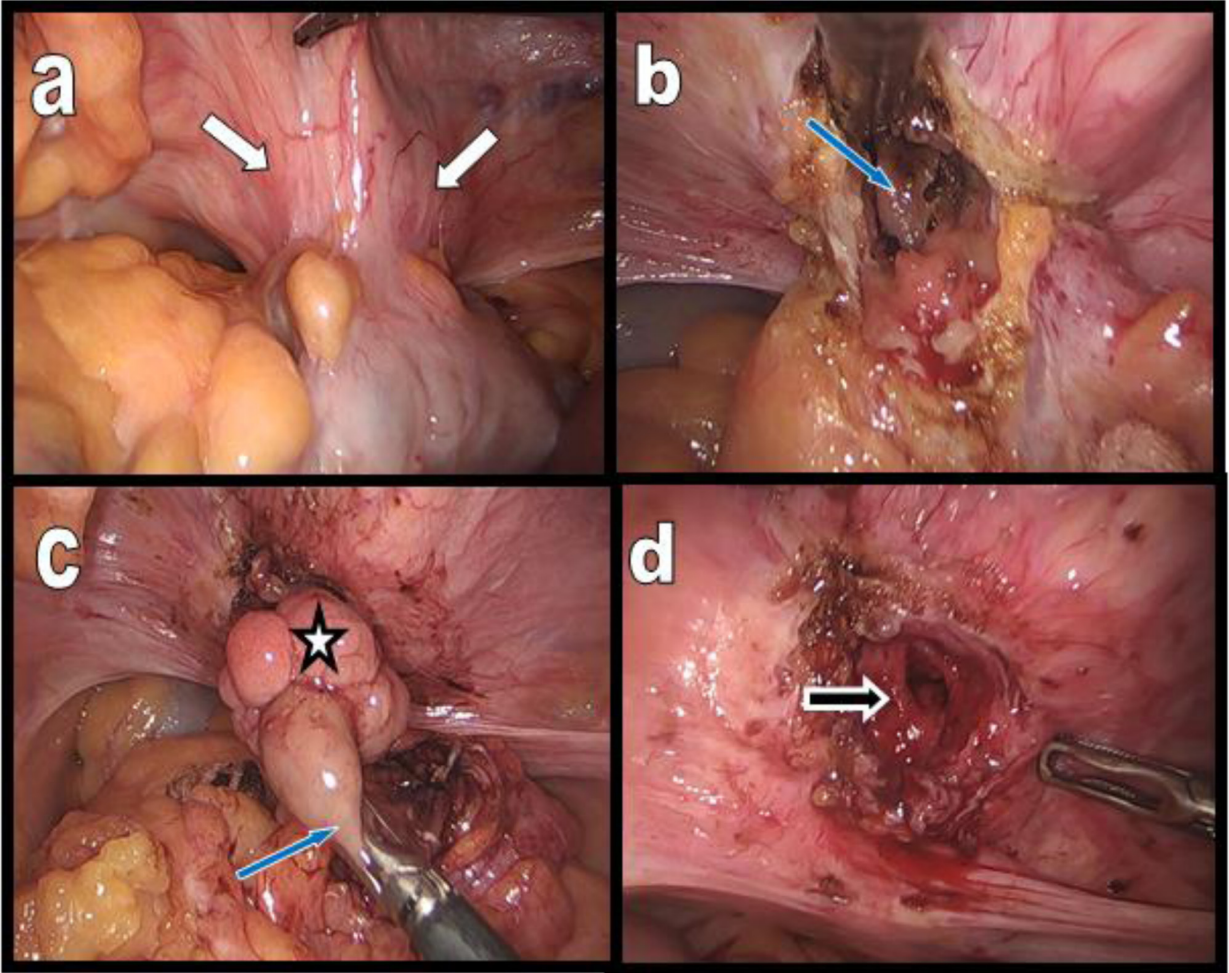


During laparoscopy (Figure 2a–d) a large chronic synechia that had developed between the sigmoid and the bladder (white arrow) was carefully dissected revealing the fistula complex, at the bottom of which appeared an elongated structure (blue arrow). A pull on this structure led to the extraction of a 1.5 cm pedunculated polyp that had incarcerated within the bladder through the fistula. The polyp explained the unusual tumoral image protruding into the bladder. The bladder was sutured and complementary segmental sigmoidectomy was performed. The post-operative period was uneventful. Histopathology confirmed a long tubular-pedunculated adenoma (peduncle of 3.5 cm) with low-grade dysplasia and rare foci of in situ adenocarcinoma developing proximally to the colo-vesical fistulous complex.

## Comment

Colon diverticular disease may be complicated by fistulas in 4 to 20% of cases, among which colo-vesical fistulas (CVFs) are the most common (65%) [1]. The pathogenesis consists of direct extension of a ruptured diverticulum or of an erosion of a diverticular abscess into the bladder. CVFs are essentially found in the elderly. Due to the interposition and protective role of the uterus in women, CVFs are more common in males or in women after hysterectomy. The fistulous tract is commonly single, but multiple tracts are found in 8% of patients.

The clinical diagnosis is based on a bundle of clinical signs comprising pneumaturia, fecaluria, and recurrent urinary tract infections with mixed organisms, abdominal pain and rarely hematuria. Nevertheless, the condition may also be nearly asymptomatic.

CT is the standard diagnostic imaging modality owing to its high sensitivity (over 90%). Fistulas may be confirmed by the presence of gas or contrast in the bladder and/or local colonic and bladder wall thickening. The treatment of colonic diverticular CVF is surgical and the classical approach consists of dissection of the fistula, followed by excision of the involved diverticular segment. The bladder component of the CVF can be simply sutured.

To the best of our knowledge the intravesical protrusion of a pedunculated colonic polyp through a CVF has never been reported before. An implantation of the polyp just upstream of the fistulous complex had probably favored its protrusion during contractions of evacuation of the sigmoid.
